# Severus Iatrosophista, Theodosius the Philosopher and Jacobus Psychrestos, introducing Colchicum as an innovative treatment for podagra in the early Byzantine period

**DOI:** 10.31138/mjr.28.2.106

**Published:** 2017-06-27

**Authors:** Gregory Tsoucalas, Markos Sgantzos

**Affiliations:** 1 Faculty of Medicine; 2 Department of Anatomy, University of Thessaly, Larissa, Greece

**Keywords:** Colchicum autumnale, ermodaktylon, podagra, gout, Severus Iatrosophista, Theodosius the Philosopher, Jacobus Psychrestos, Byzantium

## Abstract

During the early Byzantine period, the therapeutic herb “Colchicum autumnale”, or “ermodaktylon” was introduced in the treatment of podagra (gout). Podagra presented throughout the Byzantine period a disease with high incidence, since 14 out of the total 86 Emperors seem to have suffered from it. The lead pipes of the city of Constantinople’s sewer system, utensils, but also the production of the sweetening grape syrup sapa contributed to its appearance. Although Alexander of Tralles considered to be the physician who discovered the properties of the plant, Severus Iatrosophista, Theodosius the Philosopher and Jacobus Psychrestos, were the healers who introduced ermodaktylon as the pioneering treatment of podagra in the early Byzantine period.

## INTRODUCTION

**Figure 1:**
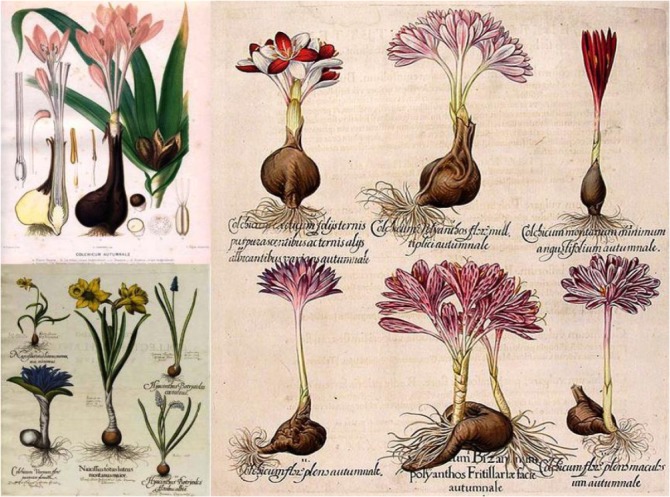
Colchicum, chromolithograph by Portail, Auguste Faguet, Dictionnaire de botanique by Henri Ernest Baillon, volume 3, 1876–1892 (left top side). Hand coloured engraving, Colchicum autumnale Luteum, Hortus Eystettensis, Basilius Bessler, 1620 (left bottom side). Hand coloured engraving: Double-flowered colchicum, Striped double-flowered colchicum, Multiflorous two-toned colchicum, Multiflorous colchicum, Small alpine colchicum, Hortus Eystettensis, Basilius Bessler, 1613 (right side).

**Figure 2:**
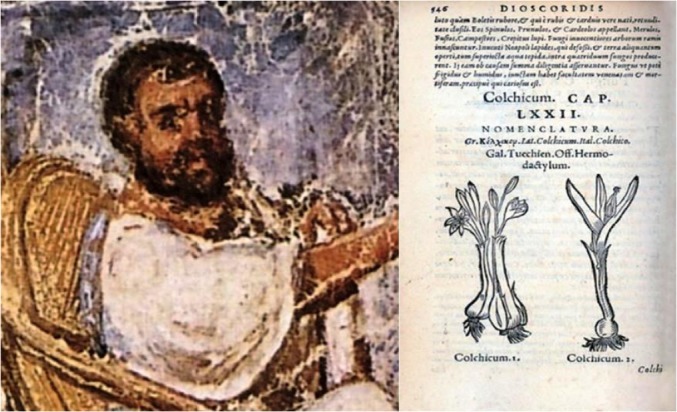
Pedanius Dioscorides, coloured lithograph, portrait, Der Wiener Dioskurides: Codex medicus Graecus 1 der Österreichischen Nationalbibliothek, Graz: Akademische Druck, Vienna (left side). Cholchicum, Dioscorides De materia medica, Bebelius, Basel, 1529 (right side).

**Figure 3:**
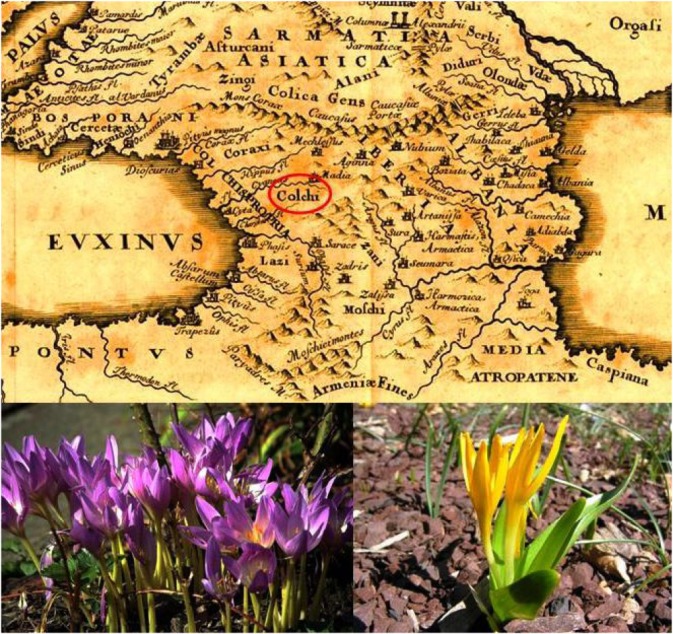
Map of Colchis and Iberia by Christoph Cellarius printed in Leipzig in 1706 (top side). Colchicum autumnale and Colchicum luteum, endemic plants in Asia Minor, Colchis (bottom side).

Podagra (from the Greek words “pous,” meaning “foot”, and “agra,” meaning “seizure” - gout) disease, a complex form of arthritis currently known as gout, was apparently accumulated among Byzantine Emperors, and common citizens of Constantinople. It has been considered that gout may be a disease caused by lead poisoning: a contributing cause for this accumulation may have been exposure to high levels of lead, originating from Constantinople’s water pipes, wine containers and cooking pots used for producing the sweetening grape syrup sapa (Greek: έψημα). Hippocrates (ca 460-370 BC), 10 eons earlier, suggested that podagra (Greek: ποδάγρα) was the result of the excess-accumulation of the body humours (4 humours theory) inside the articular capsule of the joints. The disease was called “podagra”, meaning in Greek severe pain grabbing the leg. As podagra seems to have been a significant, widespread and invalidating disease at the era, a disease that was prevalent in Byzantium, but also a well-known entity in ancient Greece. ^1–3^ Colchicine, the alkaloid of Colchicum autumnale is currently used for the treatment of gout. Colchicum autumnale (Greek: κολχικό του φθινοπώρου), or bitter hermodactyl (Greek: ερμοδάκτυλον, the finger of Hermes), or cochlicon (Greek: κοχλικόν), or articulorum (the soul of the joints), later named as saffron, or autumn crocus, was most probably known since the early rhizotomi (Greek: ριζο-τόμοι), the pharmacobotanists practicing herbal medicine since the Prehippocratic era ( **Figure 1** ). Although it was Pedanius Dioscorides (ca 1st century AD) who firstly mentioned Colchicum as a therapeutic plant with strong poisonous action ( **Figure 2** ), it was Alexander of Tralles (Greek: Αλέξανδρος ο Τραλλειανός) (525–605 AD) who was wrongfully credited with having introduced it intothe treatment of gout. ^4–7^ He himself, mentioned Severus Iatrosophista, Theophilus the Philosopher, and Jacobus Psychrestos who were the first healers to use the plant Colchicum. Although Alexander had proposed the use of cantharides as a blister to treat podagra, he had adopted previous knowledge to emphasise on a new promising herbal treatment. Alexander gave us six recipes containing hermodactyl; the final two of which he had attributed to other physicians: one to Jacobus Psychrestus, and another to Theodosius the Philosopher. ^8^ The plant’s name comes from Colchis, a region in Asia Minor, were Colchicum was endemic at the era ( **Figure 3** ). ^9^

Our historical note presents the first Byzantine physicians who introduced Colchicum autumnale as a treatment for podagra. Although Colchicum autumnale is no longer recommended due to its significant toxicity, its active components, colchicine in particular, are still widely used. The plant contains colchicine (0.8% in the seed; 0.6% in the corm), and smaller amounts of secondary alkaloids, such as demecolcine. German botanist Hyeronimus Bock (Latinized: Tragus) (1498–1554 AD) mentioned in his treatise “Kreuterbuch” during 1550, that Arab and Byzantine physicians employed the plant in the early Byzantine period. ^10^

## SEVERUS IATROSOPHISTA, THEODOSIUS THE PHILOSOPHER

Severus Iatrosophista (Greek: Σευήρος ο Ιατροσοφιστής), was a 5th century medicophilosopher, known for his masterpiece “De Clysteribus” (Greek: Περί Ενετήρων); suggesting clysters for various diseases such as skin rash. He was the first Byzantine physician to describe the allergic shock from food and drugs, while he had noted women’s hysteria proposing acute intervention. He had also presented a thorough report upon the surgical instruments availliable in that period. He is considered by Alexander of Tralles the first to introduce hermodactyl (Colchicum). ^8,11^ Theodosius the Philosopher, an eminent scholar of the same era, had also proposed the same plant as a botanic cure against podagra. ^3^

## JACOBUS PSYCHRESTOS

**Figure 4:**
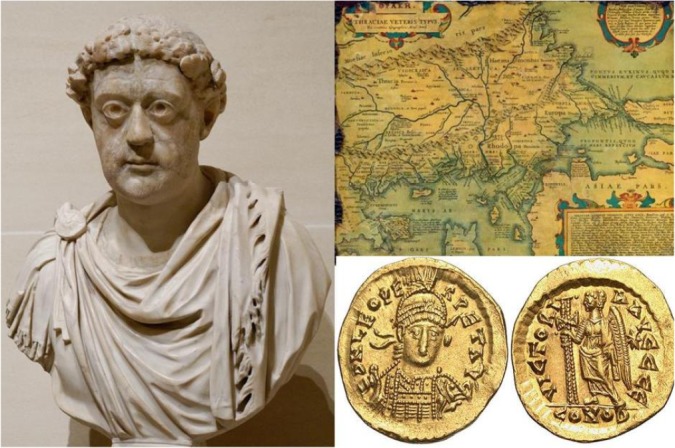
Imperial portrait of Leo the 1st, Louvre Museum (left side). Ancient map of Thrace made by Abraham Ortelius in 1585, stating both the names Thrace and Europe (right side top). Gold solidus of Leo the 1st, struck between 462–473 AD at Constantinople (right side bottom).

The Byzantine physician Jacobus Psychrestos (Greek: Ιάκωβος Ψυχριστής, or Ψυχρηστός, or Ιάκωβος Κίλιξ), was the offspring of Hesychios from Damascus, a known physician of the era. He had exercised both medicine and philosophy in Constantinople during the 5th century AD. He was considered an authority (Greek: άριστος), capable to treat a plethora of diseases. He had soon become the personal head physician (Greek: αρχίατρος) of the Byzantine emperor Leo the 1st, known as the Thracian (Greek: Λέων Α’ ὁ Θρᾷξ) (401–474 AD) ( **Figure 4** ). ^12^ Jacobus studied podagra, « τας ζεούσας φλεγμονάς των ποδών » (seethe inflammation of the legs, and proposed an innovative treatment, the Colchicum autumnale, re-introducing Severus’ and Theodosius’ suggestions. He had recommended, a viscid plaster made by the herb (Greek: βότανο), to be put upon the inflamed joint. ^8,13–14^ Today it is known that the plants of the genus Colchicum contain colchicine, a toxic natural product and secondary metabolite, which is still in use for the treatment of gout. ^2^ Jacobus was a scholar with a broad spectrum of knowledge concerning human physiology. Thus, he was also considered as an exceptional dietician, proposing a moistens temperate diet (Greek: υγραίνουσα εύκρατον δίαιτα) for the organism’s homeostasis to be balanced. ^15^ Although Jacobus was an eminent physician in the imperial court of the Byzantine empire, his work was lost, and only fragments survived inside the treatises of other important medical figures such as Damascius the philosopher (458–550 AD), Alexander of Tralles, and Aetius of Amida (ca mid 5th-mid 6th century AD). ^13,15,16^ His pioneering thought to use colchicine to treat podagra should grant him a place among the significant figures of the history of rheumatology.

## DISCUSSION - EPILOGUE

**Figure 5:**
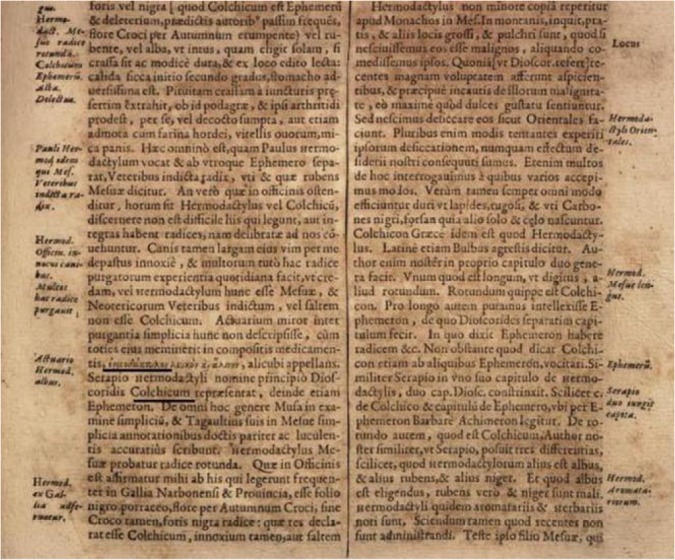
The term “hermodactyl” in Hellenic language (Greek: ερμοδάκτυλον), or Colchicum, Bauhino I. Historia plantarum universalis, nova, et absolutissima, cum consensu et dissensu circa eas. Ebroduni, Helvetia, 1651.

It appears that a great number of Sovereigns (14 of the 86 Byzantine Emperors) of the Byzantine Empire and officials of the State and leaders of the Church suffered from what in most cases seems to have been the podagra disease. The texts of the most Byzantine writers referred to the main medicine for arthritis during that era, hermodactylus, a constituent of the herb Colchicum autumnale. Hermodactylus means, in the Hellenic language, the “finger of Hermes”; the Olympian messenger god of the ancient Greeks ( **Figure 5** ), thus, perhaps, suggesting the speed of cure that the herbal drug provided for the patients. Although by many researchers Alexander of Tralles is considered to be the physician who introduced this drug in the treatment of podagra, it was the personal physician of Emperor Leon the 1st, Jacob Psychrestus, who epitomized Severus’ and Theodosius’ proposals and systematized its use; quickly gaining substantial fame.

In the history of medicine, a plethora of forgotten physicians, such as our three Byzantine healers, still seek their rightful place in medical history. Rheumatology owes them an honourable citation.
